# Will multimodal large language models ever achieve deep understanding of the world?

**DOI:** 10.3389/fnsys.2025.1683133

**Published:** 2025-11-17

**Authors:** Igor Farkaš, Michal Vavrečka, Stefan Wermter

**Affiliations:** 1 Department of Applied Informatics, Comenius University Bratislava, Bratislava, Slovakia; 2 Czech Institute of Informatics, Robotics and Cybernetics, Prague, Czechia; 3 Department of Informatics, University of Hamburg, Hamburg, Germany

**Keywords:** symbol grounding problem, embodied cognition, large language model, modalities, integration, development

## Abstract

Despite impressive performance in various tasks, large language models (LLMs) are subject to the symbol grounding problem, so from the cognitive science perspective, one can argue that they are merely statistics-driven distributional models without a deeper understanding. Modern multimodal versions of LLMs (MLLMs) are trying to avoid this problem by linking language knowledge with other modalities such as vision (Vision Language Models called VLM) or action (Vision Language Action Models called VLA) when, for instance, a robotic agent, is acting in the world. If eventually successful, MLLMs could be taken as pathway for symbol grounding. In this work, we explore the extent to which MLLMs integrated with embodied agents can achieve such grounded understanding through interaction with the physical world. We argue that closing the gap between symbolic tokens, neural representations, and embodied experience will require deeper developmental integration of continuous sensory data, goal-directed behavior, and adaptive neural learning in real-world environments. We raise a concern that MLLMs do not currently achieve a human-like level of deep understanding, largely because their random learning trajectory deviates significantly from human cognitive development. Humans typically acquire knowledge incrementally, building complex concepts upon simpler ones in a structured developmental progression. In contrast, MLLMs are often trained on vast, randomly ordered datasets. This non-developmental approach, which circumvents a structured simple-to-complex conceptual scaffolding, inhibits the ability to build a deep and meaningful grounded knowledge base, posing a significant challenge to achieving human-like semantic comprehension.

## 1 Introduction

Due to AI emergence (in the 1950s) and subsequent invention of more neural machine learning approaches (from 1990s), now we have generative AI that has progressed significantly over the last decade, resulting in large language models (LLMs), trained on vast amounts of text (Minaee et al., 2025). We have huge language models that can be used—when accompanied by appropriate interface modules—for various linguistic tasks (Brown et al., 2020). Since language describes the world, LLMs contain the knowledge of the world encoded in neural networks, with all its implications. Language has a strong expressive power, yet it is discrete (since words are symbols) and certain world knowledge is hard to describe in words (a picture is worth a thousand words or how to drive a car).

In the paper, we approach the concept of understanding as a graded phenomenon, that is, a matter of degree, which can be tested behaviorally. We look at understanding from the perspective of grounding that enables the learning system to acquire intrinsic meanings (semantics) autonomously (Harnad, 1990). In a nutshell, deep understanding is assumed to be human-like by definition. LLMs are argued to lack understanding because they are completely ungrounded. Current approaches to combine LLMs with various modalities are assumed to provide shallow understanding, and hence the main question is whether this path can eventually lead to human-like deep understanding.

### 1.1 Symbol grounding problem

LLMs are subject to the symbol grounding problem (SGP) (Harnad, 1990), because the meanings of the words they generate are not grounded in the world (Huang et al., 2023; Vavrečka and Farkaš, 2014). More specifically, the SGP has been further developed toward the vector grounding problem (Mollo and Millire, 2023), because in an LLM, the words are not represented as symbols, but subsymbolically as high-dimensional vectors (representing the meaning). Despite this change, the grounding problem remains because vector components are not connected to the world either but to other symbols.

As such, the representations produced by LLMs are generally still decoupled from perceptual and sensorimotor experience, keeping the system domain closed and unable to develop intrinsic meaning or intentionality (Bisk et al., 2020; Incao et al., 2024). This limitation has significant implications for applications that require an understanding of situated meaning, such as embodied AI, human–robot interaction, or multimodal perception. Even when LLMs are augmented with external tools or coupled with sensors and actuators (as in robotics), bridging the gap between symbolic tokens, neural representations, and embodied experience remains a major challenge (Tellex et al., 2020).

Recent research has proposed various strategies to mitigate this problem, such as grounding language in visual perception (Bender and Koller, 2020; Šejnová et al., 2018; Zhang et al., 2023), action (Mao et al., 2019; Szot et al., 2024), or interactive dialogue (Yu et al., 2020; Jokinen and Wilcock, 2024). However, these efforts often fall short of full grounding, since they still rely heavily on pre-trained LLMs with static linguistic priors. This means that language is first developed separately and only later it becomes linked to other modalities. Addressing the vector grounding problem may ultimately require architectures that integrate language, perception, and action in a tightly coupled developmental framework, where meaning emerges from ongoing interaction with the world (Barsalou, 2008; Cangelosi and Asada, 2022; Kerzel et al., 2023).

### 1.2 Turing test and irrationality

LLMs have passed the Turing test and have progressed impressively in various tasks (Jones and Bergen, 2025). They have even been considered candidates for Artificial General Intelligence (Bubeck et al., 2023). However, shortcomings have been identified, labeled “hallucinations” (Banerjee et al., 2024) that also involve errors of reasoning, although the newer versions of LLM are improved compared to their predecessors (Mirzadeh et al., 2025). However, LLMs still show irrationality as humans, but in different ways (Macmillan-Scott and Musolesi, 2024). When LLMs give incorrect answers to these tasks, they are often incorrect in ways that differ from human-like biases. Their errors stem from statistical pattern matching on vast text corpora without true comprehension, rather than from the evolutionary-developed cognitive biases and emotional states that influence human judgment. In addition to this, LLMs reveal an additional layer of irrationality in the significant inconsistency of responses. This key aspect of AI will likely be investigated in more depth in the near future (Macmillan-Scott and Musolesi, 2025).

Recent research has shown that these inconsistencies can span not just factual hallucinations, but also logical reasoning, moral judgments, and even self-contradiction within the same prompt or across similar prompts (Lin et al., 2021; Perez et al., 2023). Similar problems were also detected in modular multimodal grounding architectures, where an imbalanced training dataset led to logical inconsistencies in counting tasks (Šejnová et al., 2018). These inconsistencies raise questions about the reliability and interpretability of LLM-generated outputs, particularly in high-stakes applications. As such, ongoing research on alignment, calibration, and consistency is crucial to mitigate these shortcomings and to align LLMs more closely with human expectations and rational norms (Ouyang et al., 2022; Ganguli et al., 2023).

Another fundamental problem with LLMs is their vulnerability to adversarial attacks (Wang et al., 2021; Zou et al., 2023), which means that the trained model can easily be fooled with well crafted input (prompts, in the case of language) into producing irrational or wrong answers. This problem is general and applies to all deep models in various domains, be it computer vision, games, or language (Ren et al., 2020). Adversarial machine learning has become a modern research path that aims to overcome the vulnerability problem, but only time will tell whether this will offer a principled solution (Pelekis et al., 2025).

## 2 Building AI with grounded knowledge

**Figure 1:**
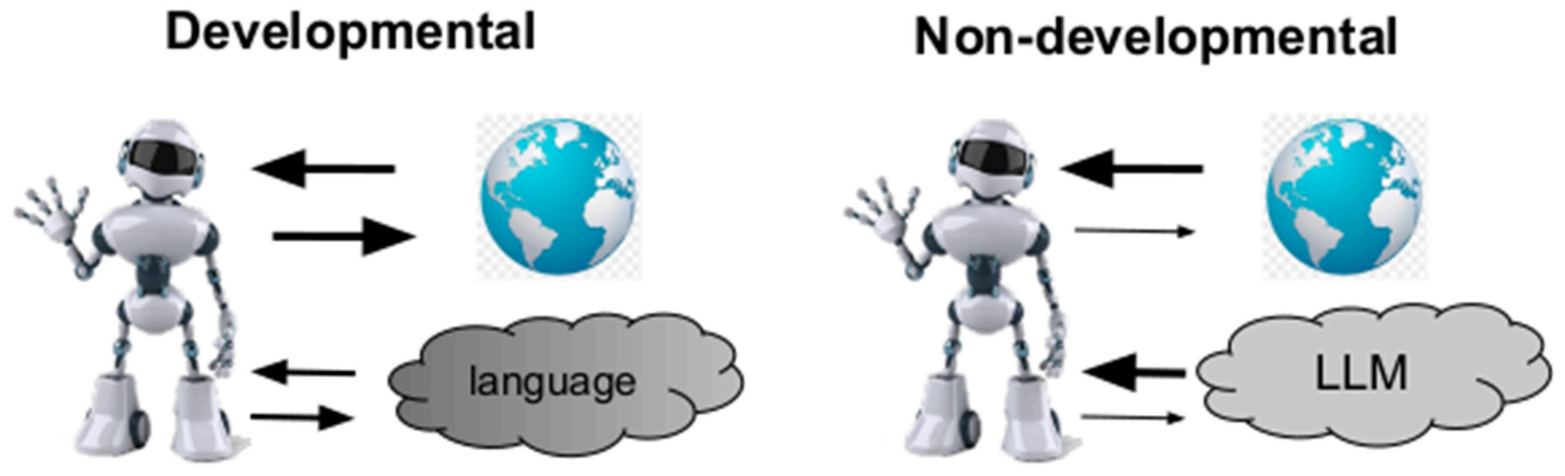
Simplified sketch contrasting two approaches to building AI agents with grounded knowledge. *Developmental approach:* Thick arrows denote rich (perception-action) interaction between the embodied agent and the multimodal world (represented by the globe). World knowledge (represented by language) is grounded gradually within developmental stages, going from more concrete toward more abstract concepts (shown by decreasing level of gray color), simultaneously with the world interaction. *Non-developmental approach:* Thick arrow from the world denotes dense information flow (represented mainly by images and videos), whereas the thin arrow denotes limited acting in the world, making the interaction very imbalanced. As for language, symbol grounding occurs in pre-given batches (using an LLM), regardless of their concreteness, with co-occurring perceptual data (from the world).

Based on recent developments in MLLMs (e.g. Allgeuer et al., 2025; Ali et al., 2025), we can see that there exist two different approaches (in several aspects) to build AI systems that could achieve a deeper understanding. We will call them developmental and non-developmental, and they are illustrated in Figure 1.

### 2.1 Developmental approach

The developmental approach is represented by the way humans acquire intelligence within their ontogeny, as also addressed in cognitive developmental robotics (Asada et al., 2009). This neurobiologically inspired approach is inspired by empirical literature showing how development progresses in stages within the so-called curriculum learning (Parisi et al., 2019) and how abstract conceptual knowledge is built on top of understanding concrete concepts in particular and reliable ways (Yee, 2019).

The developmental approach is compatible with the theories of *grounded cognition* such as conceptual metaphor theory (Lakoff and Johnson, 1980), simulation theory (Barsalou, 2008), or the action semantics framework (van Elk et al., 2013). The symbol grounding is enabled by two pathways, as argued in Reinboth and Farkaš (2022). The first pathway is direct and mainly involves action/perception, interoception, and emotions. The second pathway is indirect, being mediated by language in particular. This means, in a simplified way, that concrete words (e.g., dog, bus) are well grounded using the direct pathway, as they typically refer to objects in the world, whereas abstract words (e.g., truth, democracy) rely more on language, since they lack direct world referents. As LLM training data consists of disembodied text, which lacks the sensory, emotional, and proprioceptive inputs that form the basis of interoception, LLMs are fundamentally cut off from the direct pathway. They are involved in the indirect pathway, building complex linguistic associations, while still missing the foundational direct-grounding pathway.

In developmental robotics, the agent gradually acquires knowledge in a number of modalities (perception, action/proprioception, and language). Hence, linguistic knowledge becomes *grounded from the very beginning*^1^ and progresses during development, going hand in hand with the development of other cognitive functions. In addition, each AI agent that acquires knowledge uses its own body (single embodiment), which constrains the brain–body–world relationship.

This embodiment constraint not only provides sensory and motor contingencies, but also helps structure experience in ways that facilitate abstraction (Pfeifer and Bongard, 2007; Cangelosi and Schlesinger, 2015). Furthermore, the importance of social interaction has been widely emphasized, as it enables scaffolding, joint attention, and cultural learning mechanisms that are critical to the acquisition of language and abstract concepts (Tomasello, 1999). The developmental approach argues that truly general intelligence cannot emerge without a grounding in temporally extended experience, sensorimotor interaction, and socially mediated learning processes (Qureshi et al., 2025). This aligns with previous suggestions for the integration of developmental psychology, neuroscience, and robotics to build more human-like AI systems (Lungarella et al., 2003).

### 2.2 Non-developmental approach

The non-developmental approach is represented by modern generative AI, namely (M)LLMs. In this case, we have LLMs that have been trained on a vast number of linguistic corpora (i.e., including both concrete and abstract words) and hence are subject to the vector grounding problem. All words are treated in the same way since the distributional statistics is computed for all of them, regardless of their natural age of acquisition in humans (abstract words are typically acquired later). The non-developmental paradigm is extensively documented in a recent comprehensive survey by (Li et al. 2025a) comprising early modular, perception-driven systems to today's unified, language-centric frameworks.

While we acknowledge that modern MLLMs often employ sophisticated training strategies, including forms of curriculum learning and staged fine-tuning, our non-developmental approach refers to a more fundamental distinction. The curricula in MLLMs, however complex, are ultimately designed by humans to optimize learning on a static, pre-collected dataset and rely on passive data association. In contrast, a developmental curriculum is self-generated by the agent's own sensorimotor engagement with its environment, where learning is driven by the real-time consequences of action and exploration. The critical difference is not the presence or absence of a curriculum, but whether that curriculum relies on external passive data or it is intrinsically generated through active, embodied experience.

As a result of this difference, LLMs often fail to differentiate between the groundedness of concrete words versus abstract words, despite evidence that such distinctions are cognitively and neurally meaningful in humans (Pulvermller, 2013). Moreover, the lack of embodied interaction and temporally extended learning leads to deficits in causal and commonsense reasoning, as LLMs lack experiential continuity and episodic memory (Lake et al., 2017; Bender et al., 2021). Although these models show remarkable linguistic fluency, they remain detached from the interactive multimodal learning that characterizes human intelligence (Zador, 2019). This disconnect highlights the limitations of non-developmental systems in achieving robust generalization and true understanding.

## 3 State-of-the-art multimodal LLMs

Compared to LLM, the MLLMs process multiple modalities (text, images, audio, video, and structured data). They are trained to integrate knowledge from multiple sources and often perform very well in tasks such as visual question answering, caption generation, and cross-modal retrieval (Ge et al., 2024). Models that integrate only vision and language are called VLM and are used in tasks without motor modality (Wu et al., 2024).

Given the “passive nature” of VLMs, more recent models attempt to include the motor modality. It has a special feature, as it is related to a concrete embodiment that defines all degrees of freedom (Vemprala et al., 2024; Li et al., 2024b; Lan et al., 2025). As such, it is not trivial to generate huge collections of data to be used later for learning (Peng et al., 2024). Moreover, motor interaction datasets are often specific to particular robotic platforms and require physical execution, making large-scale, standardized motor data scarce and costly to produce. This creates a bottleneck for training general-purpose embodied agents, in contrast to the relative abundance of text or image datasets. There is a possibility to create training data for multimodal systems in simulations (Vavrečka et al., 2021; Li et al., 2024a) but they need to be fine-tuned to the real robots.

This recent category of MLLMs is represented by Vision Language Action Models (VLA) (Kim et al., 2025), which can be described as interactive systems or agents that take advantage of VLMs or MLLMs as components to perceive, reason, and act within a given environment (Šejnová et al., 2024). The newest VLA models (Zhao et al., 2025) are capable of reasoning about possible future states by adopting chain-of-thought reasoning (Zhao et al., 2024; Chen et al., 2025), wherein a model predicts helpful intermediate representations before choosing actions. These models integrate high-level planning with low-level control and are frequently designed to operate under partially observable conditions using dynamic world models. VLA systems are often embodied in humanoid robots and tested in real-world tasks, ranging from manipulation and navigation to goal-driven interaction (NVIDIA et al., 2025; Team et al., 2025). For a detailed review of VLA capabilities, see (Din et al. 2025).

The best-known VLA models include Google's RT-2 (Brohan et al., 2023), which combines VLMs with robotic control policies, and PaLM-E (Driess et al., 2023), a general-purpose embodied MLLM that processes visual, linguistic, and proprioceptive inputs. DeepMind's Gato MLLM (Reed et al., 2022) also stands out as an apparently universal agent capable of playing Atari games, captioning images, and controlling a robot arm using the same neural architecture. Other efforts like SayCan (Ahn et al., 2022) and VIMA (Jiang et al., 2023) emphasize instruction-following through natural language grounding, enabling flexible and scalable robotic behaviors. These models demonstrate the increasing capability of VLA systems to bridge perception, cognition, and action, paving the way toward more general and adaptive embodied agents. A very recent effort, NVIDIA's Cosmos-Reason1, directly tackles this grounding problem by building models specialized in physical commonsense and embodied reasoning (Azzolini et al., 2025). The approach employs a structured two-stage training process (supervised fine-tuning and reinforcement learning) on newly curated data sets that are explicitly designed around detailed ontologies of principles of the physical world.

MLLMs integrate multiple sensory inputs and excel at perception-language tasks, but they follow a non-developmental approach, relying on massive pre-training datasets without embodied interactions, leading to the vector grounding problem, where meaning is symbolically derived from other symbols. In contrast, VLA models add the motor modality, enabling agents to perceive, reason, and act in real-world environments, making them more suitable for addressing the SGP. Although VLA systems (e.g., RT-2, PaLM-E, Gato, Gr00t or Pi0) begin to incorporate elements of a developmental approach through embodiment and interaction, they still often lack the gradual, stage-wise learning, and experiential grounding characteristic of human development. As constructive feedback, (Marshall and Barron 2025) suggest that instead of focusing on scaling up transformer models [standing behind (M)LLMs], researchers should look at biological evolution and the elegant solutions it has produced to create truly autonomous agents. By learning from the efficiency and robustness of natural systems, the field of robotics can move toward the development of intelligent machines.

An alternative approach to creating representations similar to humans is based on explicit world models, which are internal predictive simulations of an environment that allow an agent to reason about causality and plan by imagining the consequences of possible actions (Ha and Schmidhuber, 2018). The primary distinction from the VLA architecture is functional and internal; While a VLA is defined by its ability to produce motor commands, a world model is an internal component used for deliberative planning by simulating what-if scenarios before acting (LeCun, 2022). Recent advances have demonstrated that MLLMs are capable of learning implicit world models directly from internet-scale video data, enabling them to generate novel, interactive, and controllable environments from a single image prompt, thus capturing a deeper understanding of environmental dynamics than a purely reactive VLA (Reid et al., 2024). On the other hand, the MLLM's understanding is confined to statistical correlations rather than a genuine grasp of cause and effect, critically limiting its ability to plan for the long term or generalize to truly novel situations.

## 4 Discussion

Our paper asks the fundamental question whether MLLMs have the potential to ever achieve understanding of the world. In other words: Using the above-mentioned non-developmental approaches, can MLLMs in principle learn causal relations, based on common sense of the world? Examples of MLLMs typically focus on capturing correlations among modalities, but correlation does not imply causation, and many causal effects are hidden from observation. (Zhu et al. 2020) identify five core domains of cognitive AI with human-like common sense (functionality, physics, intent, causality, and utility). They argue that the next generation of AI must embrace human-like common sense to solve novel tasks.

Although MLLMs show impressive pattern recognition across modalities, they struggle to infer latent variables, predict unseen consequences of actions, or distinguish cause from coincidence, skills critical to robust common sense (Li et al., 2025b). As highlighted by (Pearl and Mackenzie 2018), causal reasoning requires interventions and counterfactual thinking, both of which are still missing in current MLLMs due to their lack of embodiment and interactive experience. Models trained passively on the data cannot easily form causal mental models or simulate hypothetical scenarios. In contrast, systems with active learning, embodied exploration, and developmental grounding (as proposed in cognitive robotics) are better suited to acquire and generalize causal knowledge, because these properties support imitation of the developmental approach. Therefore, without mechanisms for intervention and iterative feedback, non-developmental MLLMs are limited in achieving true causal understanding and thus fall short of capturing the deeper structure of human-like common sense. It is acknowledged that there exist MLLMs based on reinforcement learning using human feedback (RLHF), as an early attempt toward the inclusion of human knowledge, but in those RLHF systems the feedback is usually given at a certain single time and not continually.

Despite processing various input modalities, MLLMs *lack a nonverbal world model* – an internal, structured representation of the physical and social world that exists independently of language. Their understanding is rooted in statistical associations across data modalities, but these models do not build or manipulate mental simulations of the world the way humans do. Consequently, current MLLMs *cannot truly reason without language*. Their reasoning, perception, and decision-making processes are tightly coupled to textual representations. Unlike humans, who can form visual or sensorimotor imaginations and reason through spatial or embodied experience, MLLMs rely on verbal structures even for tasks that seem inherently non-verbal. This language dependency limits their ability for intuitive physical reasoning, spatial understanding, or mental imagery and makes their cognition fundamentally symbol-based rather than grounded in perceptual-motor reality.

These limitations of MLLMs are closely related to the hypothesis of linguistic relativity which asserts that language influences cognition (Ottenheimer, 2009). The softer version of relativity suggests that language shapes thought and perception (*linguistic relativism*). MLLMs are compatible with the stronger version of linguistic relativity (*linguistic determinism*), since they are substantially based on language-based representations to reason and understand the world. Their “cognition” is hence constrained by the linguistic information they are trained on. The absence of a non-verbal world model in MLLMs restricts their ability to form flexible, language-independent concepts, or an intuitive understanding of the world.

The limitations of MLLMs are also evident when viewed through the lens of mental imagery and theories of mental representation. According to depictive theories (e.g., Kosslyn, 1994), mental imagery involves spatial, quasi-perceptual representations that resemble visual experiences, whereas descriptive theories (e.g., Pylyshyn, 2003) argue that mental representations are symbolic and language-like. MLLMs operate solely within the descriptive domain, relying on text-based processing and lacking the capacity for depictive, image-like mental representations. As a result, they are unable to simulate mental imagery or reason spatially in a perceptual sense, limiting their ability to perform tasks that humans solve through visualization, such as imagining physical transformations or mentally rotating objects.

On the other hand, given that the progress in MLLMs is very fast, it may be that improved models will be appearing in forthcoming years, narrowing the gap toward understanding. On that path, it will definitely be inevitable to go beyond learning statistical correlations to reveal causal mechanisms. This will clearly involve a stronger role for the motor modality that facilitates this process in the context of affecting the world. Maybe the well functioning MLLMs would not be body-agnostic but rather tied to a concrete body, which is an open question. At the same time, well-trained nonlinguistic modules will have to be included to take care of all reasoning that does not require language. Last but not least, perhaps sophisticated control mechanisms will be required to carefully link modalities together, loosely resembling human development (following a growing complexity).

In summary, it remains to be seen whether these fundamental problems of MLLMs can be overcome if we assume that progress will occur in nonlinguistic AI models (supporting nonlinguistic cognition and reasoning) that will be integrated with LLMs. However, it seems that stage-wise world-based grounded integration of modalities is critical for embodied acquisition of common sense, and hence deep understanding of the world (Feng et al., 2025; Szot et al., 2025).

## Data Availability

The original contributions presented in the study are included in the article/supplementary material, further inquiries can be directed to the corresponding author.

## Footnotes

1 In this context, we can ignore the difference that humans are typically exposed to spoken language, whereas LLMs work with the text, since from the current perspective, speech-to-text technology, and *vice-versa*, is becoming a solved problem.
